# 4‐phenylbutyric acid—Identity crisis; can it act as a translation inhibitor?

**DOI:** 10.1111/acel.13738

**Published:** 2022-11-14

**Authors:** Daniel Stein, Zeev Slobodnik, Benjamin Tam, Monica Einav, Barak Akabayov, Shimon Berstein, Debra Toiber

**Affiliations:** ^1^ Department of Life Sciences Ben‐Gurion University of the Negev Beer Sheva Israel; ^2^ The Zlotowski Center for Neuroscience Ben‐Gurion University of the Negev Beer Sheva Israel; ^3^ Department of Chemistry Ben‐Gurion University of the Negev Beer Sheva Israel

**Keywords:** 4PBA, chemical chaperone, protein synthesis, Proteostasis, translation inhibition, unfolded protein response

## Abstract

Loss of proteostasis can occur due to mutations, the formation of aggregates, or general deficiency in the correct translation and folding of proteins. These phenomena are commonly observed in pathologies, but most significantly, loss of proteostasis characterizes aging. This loss leads to the chronic activation of stress responses and has a generally deleterious impact on the organism. While finding molecules that can alleviate these symptoms is an important step toward solutions for these conditions, some molecules might be mischaracterized on the way. 4‐phenylbutyric acid (4PBA) is known for its role as a chemical chaperone that helps alleviate endoplasmic reticulum (ER) stress, yet a scan of the literature reveals that no biochemical or molecular experiments have shown any protein refolding capacity. Here, we show that 4PBA is a conserved weak inhibitor of mRNA translation, both in vitro and in cellular systems, and furthermore—it does not promote protein folding nor prevents aggregation. 4PBA possibly alleviates proteostatic or ER stress by inhibiting protein synthesis, allowing the cells to cope with misfolded proteins by reducing the protein load. Better understanding of 4PBA biochemical mechanisms will improve its usage in basic science and as a drug in different pathologies, also opening new venues for the treatment of different diseases.

## Graphical abstract

4PBA is a weak inhibitor of mRNA translation, and it does not directly promote protein folding nor prevents aggregation. Instead, it possibly alleviates proteostatic or ER stress by inhibiting protein synthesis, allowing the cells to cope with misfolded proteins by reducing protein load. Such a mechanism of function is already found in the cell—in the ER stress alleviation through eIF2a phosphorylation.

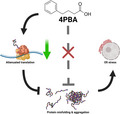

## 1 INTRODUCTION

Proteins play a critical role in the cell, and the correct folding of a protein is often performed by a specialized group of proteins—molecular chaperones—yet this is quite a delicate mission that can easily be disrupted. A protein might be mis‐ or un‐folded by suboptimal temperature, redox state, ionic balance, or other negative factors. Furthermore, the gradual loss of protein homeostasis (proteostasis) is one of the hallmarks of several pathologies and of aging (López‐Otín et al., 2013). Thus, many resources are invested in understanding this machinery and in finding possible treatments.

A possible solution is the use of chemical chaperones which help fold proteins. One such suggested—and frequently mentioned—molecule is 4‐phenylbutyric acid (4PBA). This molecule affects the cell in several ways, including histone deacetylase inhibition; as an alternative metabolite in urea cycle disorders; and in the attenuation of endoplasmic reticulum (ER) protein retention (Batshaw et al., 2001; Ma et al., 2017; Rubenstein et al., 1997; Walker, 2009). However, while its ability to alleviate ER stress is well established, there is no evidence for protein folding capacity improvement nor aggregation prevention, in any molecular or biochemical assay (Ayala et al., 2012; Luo et al., 2015; Ozcan, 2006; Zeng et al., 2017). Since 4PBA is already used in human patients and is undergoing various clinical trials (Batshaw et al., 2001; Walker, 2009), it is important to understand its effect on cells and its mechanism of action.

Our results show that 4PBA is an mRNA translation attenuator, reducing translation levels from bacteria to mammalian cells, both in vitro and in cells. However, it does not affect protein stability or refolding capacity in any of the tested substrates or methods. These results shed light on another biochemical function of 4PBA, translation attenuation, which will help to correctly use 4PBA and increase the range of its potential applications.

## 2 4PBA ATTENUATES PROTEIN TRANSLATION

**FIGURE 1:**
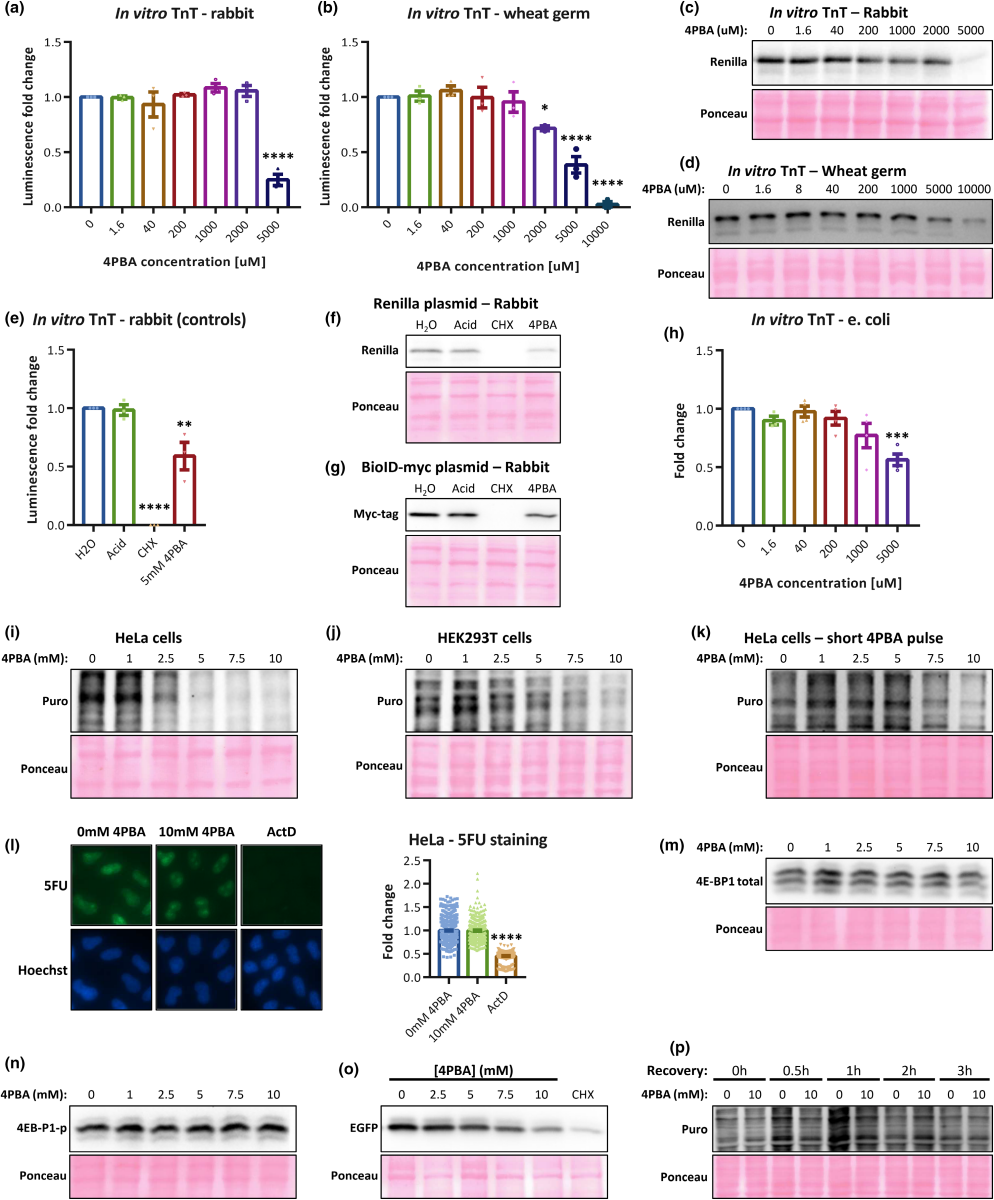
(a–d) In vitro TnT and protein blots for (a, c) rabbit reticulocyte lysate and (b, d) wheat germ. (e, f) In vitro TnT and a protein blot of a control assay showing *Renilla* expression. Acid—Water titrated to the pH of 5 mM 4PBA; CHX—Cycloheximide. (g) A representative protein blot of BioID‐myc in vitro expression. (h) Prokaryotic TnT system of firefly expression represented by luciferase activity. (i–k) Total protein translation measured using SUnSET assays in (i, k) HeLa and (j) HEK293T cells, with 4PBA treatments for (i, j) 24 h or (k) 40 min. Puro—Puromycin. (l) Transcription levels using 5‐fluorouridine (5FU) in HeLa cells with 40 min 4PBA treatment. Right panel—Quantifications of 5FU intensity. ActD—Actinomycin D (transcription inhibitor). (m, n) Total and phosphorylated 4 E‐BP1 levels in HeLa cells with 40 min 4PBA. (o) The expression of IRES‐dependent EGFP in HeLa cells, with 4PBA treatment. (p) Translation recovery from 4PBA inhibition, measured with SUnSET. Recovery—The duration of recovery with regular media, after a 1 h‐long 4PBA treatment. *p‐value < 0.05, **p‐value < 0.005, ***p‐value < 0.001, ****p‐value < 0.0001.

To characterize the 4PBA effect on proteostasis, we first measured its effect on translation. A common and sensitive method to test such effects is the use of in vitro luciferase‐based transcription‐translation systems (TnT). We used TnT kits in two eukaryotic models: Rabbit reticulocytes and wheat germ. Surprisingly, we found that 4PBA decreases the Luciferase activity in both eukaryotic systems (Figure 1a,b), in concentrations that are commonly used for its chemical chaperone activity in the literature (>1 mM) (Burrows et al., 2000; Ma et al., 2017; Rubenstein et al., 1997; Zeng et al., 2017). Protein blots verified that this reduction correlates with lower protein levels (Figure 1c,d). To confirm this is not due to the high acidic environment from the 4PBA solution, we ran the TnT assays also with HCl‐titrated water to a similar pH as of 5 mM 4PBA (marked “Acid”; Figure 1e–g). Importantly, the translation inhibition is not unique to *Renilla* (Figure 1g), and it also appears in a prokaryotic TnT system (Figure 1h).

Next, we measured protein synthesis in cellular systems using SUnSET assays in two human cell lines. Again, we found that the amount of newly synthesized proteins decreases after 24 h (Figure 1i,j). The concentrations used are often utilized in the literature to show ER stress alleviation. Moreover, 4PBA attenuates translation even after 40 min of treatment (Figure 1k), without affecting transcription (Figure 1l), nor increased 4 E‐BP1 levels or phosphorylation (Figure 1m,n). This suggests that the effect is not due to cap‐dependent signaling or transcriptional effects. Interestingly, 4PBA also affects IRES‐dependent translation (Figure 1o), pointing on global translation inhibition. The inhibition is reversible in less than 3 h after eliminating 4PBA from the medium (Figure 1p). Thus, we revealed both in vitro and in cell culture that 4PBA is a general translation attenuator, specifically in the concentrations used in previous reports.

## 3 4PBA DOES NOT AFFECT PROTEIN FOLDING IN VITRO

**FIGURE 2:**
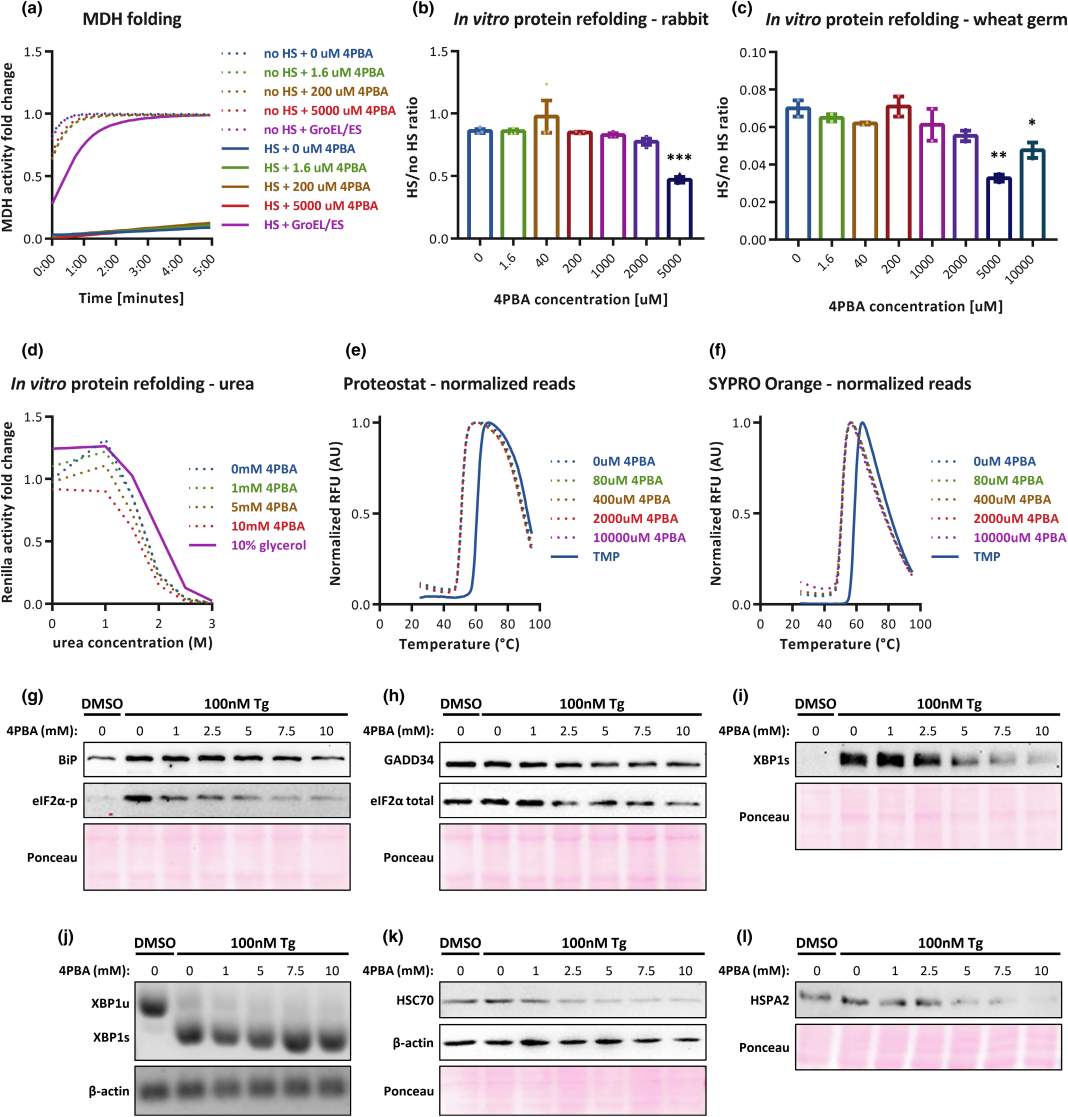
(a) A representative graph of MDH folding assays. (b, c) In vitro luciferase‐based refold assays, presenting the fraction of active *Renilla* luciferase after HS, compared with non‐heated samples (in both rabbit and wheat germ kits). (d) In vitro luciferase‐based refold assay from HEK293T lysates, presenting the *Renilla* luciferase activity after 4PBA/glycerol treatment and urea denaturation. (e, f) DHFR protein thermal shift assays with (e) Protoestat or (f) SYPRO Orange dyes. TMP—Trimethoprim (DHFR inhibitor). (g–i) Representative protein blots of the ER stress and UPR markers in different 4PBA concentrations. XBP1s—Spliced XBP1. (j) XBP1 mRNA semi‐quantitative PCR. XBP1u—Unspliced XBP1; XBP1s—Spliced XBP1. (k, l) representative protein blots of non‐ER related proteins. (g–l) the 4PBA treatment time was 8 h, out of which the later 6 h are with 100 nM Tg. *p‐value < 0.05, **p‐value < 0.005, ***p‐value < 0.001, ****p‐value < 0.0001.

4PBA was described as a chemical chaperone, but when we measured its refolding activity or prevention of heat‐induced misfolding of pig heart malate dehydrogenase (MDH), we failed to see any effect. MDH was misfolded by heat shock, and then inactive MDH was incubated with either 4PBA or Gro‐EL/Gro‐ES (chaperone) for refolding. To test protein refolding, MDH activity was measured by its substrate consumption (NADH). The bacterial chaperone system of Gro‐EL/Gro‐ES managed to refold MDH, but surprisingly, 4PBA had no effect in any of the concentrations tested (Figure 2a).

Next, *Renilla* Luciferase was expressed in the presence of different 4PBA concentrations in the TnT systems. Samples were heat‐shocked at 42°C (HS) and re‐incubated at 30°C to allow recovery and protein refolding. HS samples were normalized to their non‐HS sample (see *Experimental Procedures*). Our results reveal no increase in Luciferase activity under any 4PBA concentration, in either of the eukaryotic TnT systems used (Figure 2b,c). Note that although there seems to be a slight elevation in 10 mM 4PBA, this is an artifact due to very low Luciferase activity in both HS and non‐HS samples (Figure 1b,d, 10 mM). Using HEK293T protein lysate, we also measured 4PBA refolding from urea‐based chemical protein denaturation, and still we found no effect (Figure 2d). We conclude that 4PBA does not help to refold the tested proteins—not from heat—neither chemical‐stress environments.

Except for protein refolding, some chaperones can prevent protein aggregation and stabilize the active protein conformation. We assessed 4PBA effects in these regards using Proteostat‐ and SYPRO Orange‐based thermal shift assays and found no effect in neither of them (Figure 2e,f). Thus, 4PBA does not act as a chaperone through prevention of aggregation or by stabilizing protein structure either.

## 4 THE ER STRESS ALLEVIATION IN 4PBA TREATMENT IS DUE TO TRANSLATION ATTENUATION

Previous work has found that 4BPA treatment reduces ER stress, and it was assumed this was due to its chaperone activity. Since translation attenuation could alleviate ER stress, we hypothesized that the 4PBA rescuing phenotype is at least partially attained by reducing translation.

To test this possibility, we treated the cells with 4PBA in different concentrations and induced ER stress using thapsigargin (Tg). As expected, we found eIF2α phosphorylation levels to decrease with 4PBA, while total eIF2α levels decreased too—but to a smaller extent. This suggests that the reduction in eIF2α phosphorylation comes from ER stress alleviation and not merely from reduction in eIF2α protein levels. In addition, BiP and GADD34 levels were reduced (Figure 2g,h). Spliced XBP1 protein levels are also reduced with increasing 4PBA concentrations (Figure 2i) while the mRNA levels or splicing do not change in the 8 hr period we used (Figure 2j), indicating a translation‐based ER stress alleviation. Outside the ER, we saw that the levels of the cytosolic chaperones HSC70 and HSPA2 as well as β‐actin are reduced in higher 4PBA concentrations (Figure 2k,l). These data indicate that translation of both ER and cytosolic mRNAs is attenuated, supporting the global translation inhibition observed in the SUnSET assays (Figure 1).

## 5 DISCUSSION

Protein folding is a very delicate process, which can be very easily disrupted. Unfortunately, loss of proteostasis becomes a great problem in aging and in numerous pathologies, stressing the importance in finding proteostasis‐alleviating agents. One of these agents is 4PBA, which is well known to alleviate at least one aspect of proteostasis—ER stress. 4PBA increases the protein shuttling from the ER, thus alleviating the load of ER‐resident proteins (Burrows et al., 2000; Ma et al., 2017; Rubenstein et al., 1997). However, the required 4PBA concentrations are relatively high (1‐10 mM), probably due to its rapid metabolism (through mitochondrial β‐oxidation) which reduces its cellular concentrations. The usage in such high concentrations might lead to additional effects, some of which can act on ER stress as well.

Here, we revealed—both in vitro and in cells—that 4PBA is a weak mRNA‐translation inhibitor that reduces the load on proteostasis and quality control machinery, probably allowing a larger portion of translated proteins to be correctly folded. Interestingly, this mechanism is already found in the ER stress Unfolded protein response (UPR): the phosphorylation of eIF2α by PERK prevents new protein synthesis, reducing ER stress (Harding et al., 1999). Moreover, while suggestive by its “chaperone” title, 4PBA does not help the refolding of mis‐ or un‐folded proteins we tested, nor prevents aggregation. Importantly, we are not the first to fail in seeing chaperone activity in 4PBA treatment (Mai et al., 2018). Thus, we conclude that the ER stress alleviation upon 4PBA treatment partially stands on the same ground as the UPR—curbing mRNA translation.

4PBA is also a known pan‐histone deacetylase inhibitor (Lea et al., 1999); therefore, increased transcription—which may lead to increased translation—is expected. Here, we saw that the translation attenuation precedes any effect on transcription, leaving the relationship between hyper‐transcription and translation attenuation an important question that should be further assessed.

To conclude, as an FDA‐approved drug, better characterizing 4PBA biochemical functions would improve the ability to use it as a treatment in different pathologies and increase the understanding of the mechanism in currently treated pathologies.

## 6 EXPERIMENTAL PROCEDURES

### 6.1 In vitro protein translation and refolding assays

A 4PBA stock was freshly prepared before each experiment, by dissolving 4PBA powder (P21005‐25G, Sigma Aldrich) in ultra‐pure water (002321773100, Bio‐Lab) to a final concentration of 31.25 mM (5.13 mg/ml), then incubated at 42°C for 60–90 min, 1500RPM shaking. This stock later served as the 5 mM 4PBA treatment, therefore, the rest of the concentrations were diluted in ultra‐pure water relatively. To avoid precipitation of the powder, all dilutions were done while at 42°C incubation. Alternatively, we also ran the assay using 31.25 mM 4PBA stock that was titrated with an equimolar amount of NaOH and found very similar results to the heat‐dissolved 4PBA stock.

Promega Rabbit‐based TnT® T7 Quick Coupled Transcription/Translation System kit (L1170, Promega) was used according to the manufacturer's protocol. Briefly, each sample contained 40 μl TnT reaction mix, 1 μl Met (1 mM), 1 μl T7‐*Renilla* Luciferase plasmid (1 μg/μl), and 8 μl of the treatment stock (e.g., 31.25 mM 4PBA solution, for the 5 mM 4PBA sample). Samples were incubated at 30°C for 20 min, and translation was arrested by adding 1 μl CHX (10 mg/ml) to a final concentration of ~0.2 mg/ml (to prevent possible artifacts due to de novo protein synthesis). Then, 25 μl of each sample were transferred to a new tube (labelled as: “heat shock”), the original tubes were re‐incubated at 30°C and the heat shock tubes at 42°C, for 15 min. Once heat shock ended, all tubes were re‐incubated at 30°C for 20 min, for protein refolding.

To obtain the Luciferase luminescence reads, Promega *Renilla* Luciferase kit (E2810, Promega) was used according to the manufacturer's protocol, using 5 μl of each TnT sample (both heat‐shocked and non‐heat‐shocked samples). Protein refold capacity was measured as the ratio between HS and no HS reads.

Protein samples for western blots were prepared by mixing 15 μl of the TnT samples with 15 μl water and 10 μl homemade Laemmli X4 buffer.

Wheat germ‐based experiments were done similarly with adaptation to the kit protocol, with this minor difference in 4PBA stock preparation: instead of 31.25 mM stock, a 25 mM stock was prepared. Myc‐tag TnT assay was done with the Rabbit reticulocytes TnT kit, and with a BioID‐myc‐tag plasmid instead of the T7‐*Renilla* plasmid.

Acidity control assays (Figure 1e–g) were done in the Rabbit‐based TnT kit, with either regular H_2_O (pH ~7), acidic H_2_O (by titration with HCl to a similar pH of the 5 mM 4PBA), cycloheximide (100 μg/ml) or 5 mM 4PBA.

### 6.2 E. coli‐based in vitro translation assay

The E. coli cell‐free extract was made as previously described with some modifications (Kim et al., 2006). The cell extracts were prepared from E. coli strain BL21 Rosseta2 (DE3). The cells were grown at 37°C in 3 (1L media in 3L Erlenmeyer) of 2xYT (yeast extract 10 g/L, Tryptone 16 g/L, NaCl 5 g/L, pH 7) at 220 RPM. When the cell density (OD600) reached 0.6, isopropyl‐thiogalactopyranoside (IPTG), 1 mM was added to the cell culture media to express T7 RNA polymerase. The cells were grown for 4 h and washed three times by suspending them in 500 ml of buffer A and centrifuged. Buffer A (10 mM Tris–acetate buffer (pH 8.2), 14 mM magnesium acetate, 60 mM potassium glutamate, and 1 mM dithiothreitol (DTT) containing 0.05% (v/v) 2‐mercaptoethanol (2‐ME). Before storing the pellets at −80°C, the wet cell pellets were weighed. For each 10 g of thawed cells, 12.7 ml of buffer B were added (buffer A without 2‐ME) and underwent lysis by high pressure homogenizer at a constant pressure of 15,000 psi. The lysate was then centrifuged at 14,000 *g* RCF for 45 min at 4°C, and the top layer of the supernatant (lipid layer) and pellet were carefully removed and then centrifuged again. The lysis was incubated for 90 min, 370RPM. The sample was centrifuged again at 10,000 *g* RCF for 10 min at 4°C and the pallet discarded. The extract was dialyzed against Buffer C 10 mM HEPES‐NaOH [pH 7.4], 10 mM NH4Cl, 60 mM K‐glu, 14 mM MgCl2, 4 mM DTT) in 40 for 4 h and centrifuged again at 21,000 RCF for 15 min at 4°C and the pellet removed; the extract aliquot was frozen at −80.

The transcription/translation assay was performed as previously described with some modifications (Harding et al., 1999). The inhibition effect of 4PBA on E. coli translation was tested. The *Renilla* Luciferase was used as a reporter gene for translational activity. The reaction mixture contained: 56 mM HEPES‐KOH (pH 7.5), 3.9% polyethylene glycol 8000, 0.08 mg/ml tyrosine, 1.1 mM ATP, 0.75 mM for CTP, GTP and UTP, 180 mM potassium glutamate, 72 mM creatine phosphate, 25 mM ammonium acetate, 0.58 mM cAMP, 1.5 mM DTT, 0.031 mg/mL folinic acid, 0.151 mg/ml E. coli tRNA mix, 1 mM of each amino acid, 20 mM magnesium chloride, 0.25 mg/mL creatine kinase, 0.02 mg/ml T7 RNA polymerase, E. coli cell‐free extract was 25% of the reaction volume, 14 ng/μl *Renilla* Luciferase‐encoding plasmid and the tested molecule in concentrations ranging from 1.6 μM to 5 mM.

The reaction mixture was incubated at 37°C for 1 h and terminated by the addition of 1.6 reaction volume of 8 μM erythromycin at a final concentration of 5 μM. The reactions were transferred to white 96 well plate and the result measured by a plate reader (Synergy H1 BioTek). *Renilla* luciferin assay reagent (*Renilla*‐LAR, Promega) was added at 3:2 (*Renilla*‐LAR: reaction). *Renilla*‐LAR was injected into each well followed by a luminescence read.

### 6.3 Cell cultures

All cells were grown in DMEM (catalog number: 41965039, Thermo‐Fisher Gibco), supplemented with 1% L‐glutamine (catalog number: 25030024, Thermo‐Fisher Gibco), 1% Penicillin/Streptomycin antibiotics mix (catalog number: 15140122, Thermo‐Fisher Gibco) and 10% FBS (catalog number: 12657‐029, Thermo‐Fisher Gibco). Cells were incubated in a humid 37°C incubator with 5% CO_2_.

### 6.4 4PBA SUnSET assays in cells

To treat cells, 10 mM 4PBA medium was freshly prepared before each experiment by adding 4PBA powder (P21005‐25G, Sigma Aldrich) to medium (see *“Cell Cultures”* section). Then, to achieve full dissolution, the media were titrated with an equimolar amount of NaOH and heated in 37°C, 300RPM shaking until full dissolution (less than one hour). Alternatively, the non‐titrated 4PBA medium can be heated in 42°C for 1–2 h with occasional mixing, to achieve full dissolution. Both techniques gave very similar results. Regular medium (0 mM 4PBA) was also incubated and treated simultaneously (excluding titrations). The rest of 4PBA concentrations were achieved by different dilution ratios between the 0 mM and 10 mM 4PBA media. The long 4PBA treatments in cells (Figure 1i,j) were 48‐hour long, and the media were changed to freshly prepared ones after 24 h. The short 4PBA treatments (Figure 1k) were 10‐min long, followed by a puromycin pulse, leading to a total of 40 min of 4PBA treatment, out of which 30 min with puromycin.

The SUnSET(Schmidt et al., 2009) experiment was done by adding puromycin at a final concentration of 10 μg/ml directly to the cell media, followed by 37°C incubation for ~15 to 30 min in HeLa cells and ~10 min in HEK293T cells. Once puromycin labelling was done, cells were collected for total protein extraction.

The 4PBA recovery SUnSET assay (Figure 1l) was done by incubating cells with 0/10 mM 4PBA media for 1 h, then media were changed to non‐4PBA media for the indicated recovery period. The 10 ug/ml puromycin pulse was in the last 25 min of the recovery time.

### 6.5 5‐Fluorouridine incorporation in HeLa cells and immunofluorescence

Cells were plated in an 8‐well micro‐slide (catalog number: 80826, ibidi), 8000 cells per well. On the following day, media of 0/10 mM 4PBA concentrations were prepared as mentioned above (see *“4PBA SUnSET assays in cells”* section), and cells were treated with 0 mM 4PBA, 10 mM 4PBA or 0.6 μg/ml Actinomycin D (“ActD”; catalog number: A‐4262, Sigma‐Aldrich) for 40 min. 10 min after treatment initiation, 5‐Fluorouridine (“5FU”; catalog number: 329371, Sigma‐Aldrich) was added directly into each well, to a final concentration of 2.5 mM. In total, cells were treated for 40 min with 4PBA/ActD, out of which 30 min were with 5FU. Additional critical controls were 0 min 5FU (background staining) and 40/60 min of 5FU (to verify the experimental wells did not reach a saturated 5FU staining). These data are not shown.

Once 5FU labelling reached the end, cells were washed with PBS, fixated with 4% paraformaldehyde, washed with PBS and permeabilized (5 min with 0.1% sodium citrate, 0.1% Triton X‐100, pH 6). Each well was then washed 3 times in washing solution (0.25% BSA, 0.1% Tween‐20 in PBS), blocked for 30 min with blocking solution (10% goat serum and 90% washing solution) and incubated overnight in 4°C with anti‐BrdU (diluted 1:300 in blocking solution; catalog number: B8434, Sigma‐Aldrich). On the following day, each well was washed 3 times with washing solution, then incubated 60 min in room temperature with anti‐mouse AF488 (diluted 1:200 in blocking solution; catalog number: A32723, Invitrogen), then washed again twice with washing solution. For nuclear staining, the wells were incubated 5 min in room temperature with Hoechst 33342 (10ug/ml; catalog number: B2261, Sigma‐Aldrich), then washed twice with PBS.

After the acquisition of the fluorescent images, they were automatically analyzed for mean GFP fluorescence intensity using CellProfiler 4 (Stirling et al., 2021).

### 6.6 4PBA total protein samples in HeLa cells

For the 4 E‐BP1 (total/phosphorylation) protein samples, media of different 4PBA concentrations were prepared as mentioned above (see *“4PBA SUnSET assays in cells”* section). Then, similar to the short 4PBA treatment SUnSET assays, medium was changed to the corresponding 4PBA treatment for 40 min, after which cells were collected for total protein extraction.

For the IRES‐EGFP protein samples, cells were transfected with IRES‐EGFP plasmid (kindly donated by Prof. Ran Taube). Then, media of different 4PBA concentrations were prepared as mentioned above (see *“4PBA SUnSET assays in cells”* section), and cells were treated with 4PBA or cycloheximide (“CHX”, 1ug/ml; catalog number: 01810, Sigma‐Aldrich) 3–4 h after transfections. Cells were then collected for total protein extraction 5–6 h after 4PBA treatment started (a total of 8–9 h after transfections).

### 6.7 MDH folding assay

Mammalian mitochondrial malate dehydrogenase (MDH) from pig heart was incubated in a dry bath at 47° for 30 min to achieve complete unfolding (control tube was incubated on ice). Inactivation mix contained: 50 mM triethanolamine, 40 mM MgAc_2_, 300 mM KCl, 1 mM DTT, 600 nM MDH, H_2_O. After unfolding, a refolding mix was added (50 mM triethanolamine, 4 mM ATP, 1 mM DTT, 20 U/ml PK, 20 mM PEP, H_2_O) with the correct chaperone treatment, at a 1:1 ratio. For GroEL/GroES controls, 600 nM GroEL and 1.2 μM GroES were added. Mixed samples were incubated for 30 min in 37°C (control tube was incubated on ice). 20 μl from each reaction was transferred to a well with 180 μl of MDH activity buffer (165 mM K‐phospate pH 7.4, 1.1 mM DTT, 550 μM oxaloacetate, 330 μM NADH). Change in 340 nM absorbance was measured every 6–10 s for 5 min.

In addition, this experiment was also done with additional concentrations: 0, 50, 200, 500, 1000, 5000 μM 4PBA. These concentrations did not have any effect on MDH activity either, in any of the biological nor technical replicates. The data of this experiment are excluded because of lack of space.

### 6.8 In vitro protein refolding—urea denaturation

200k–400k HEK293T cells were plated in a 60‐mm cell culture plate. On the following day, cells were transfected with T7‐*Renilla* Luciferase plasmid and left for 24–72 h to express the Luciferase. Then, they were collected and lysed using 750 μl Promega Passive Lysis Buffer X1 (“PLB”, catalog number: E194A, Promega), supplemented with 100 μg/ml cycloheximide (“CHX”, catalog number: 01810, Sigma‐Aldrich), according to the PLB protocol. Once lysis was done, lysate was centrifuged for 3 min in 17,000 *g* and supernatant was transferred to a new tube (termed “clear Luciferase”).

20 mM 4PBA stock was prepared by dissolving 4PBA powder into PLB and titrating with an equimolar amount of NaOH. The 20 mM 4PBA was diluted in PLB to the following stock concentrations: 20, 10, 2, 0 mM, and in addition a 20% glycerol (in PLB) was prepared.

The clear Luciferase was pre‐treated with the tested chaperones (4PBA/glycerol) by mixing 130 μl clear Luciferase with 390 μl of each of the above‐mentioned 4PBA/glycerol stocks and incubated for 30 min at 30°C, 250–350 RPM. The concentrations in these tubes: 15, 7.5, 1.5, 0 mM, and in addition 15% glycerol.

To prepare the urea denaturants, 9 M urea powder was dissolved in PLB, and diluted (with PLB) to the following concentrations: 9, 7.5, 6, 4.5, 3, 0 M.

To denature the Luciferase, 80 μl of each chaperone pre‐treated Luciferase sample were mixed with 40 μl of each urea sample (leading to a matrix with 30 different chaperone‐urea combinations). All tubes were incubated again for 30 min at 30°C, 250–350 RPM. The final 4PBA concentrations are: 10, 5, 1, 0 mM and 10% glycerol; the final urea concentrations are: 3, 2.5, 2, 1.5, 1, 0 M.

The *Renilla* Luciferase activity was then measured using Promega *Renilla* Luciferase kit (E2810, Promega), as required by the manufacturer. Relative luminescence reads (RLUs) were normalized to the 0 mM 4PBA, 0 M urea sample.

### 6.9 Proteostat thermal shift aggregation assay

To measure protection against protein aggregation, 4PBA was tested in a thermal shift assay using Proteostat (catalog number: ENZ‐51023, Enzo). 16.67 mM 4PBA initial stock was prepared by diluting 4PBA powder in Enzo assay buffer X1 and titrating with an equimolar amount of NaOH. Then, the initial stock was diluted to the following concentrations: 16.67, 3.33, 0.67, 0.13, 0 mM. trimethoprim (TMP) is an inhibitor of DHFR and was used as a positive control (diluted in 0 mM Enzo assay buffer X1).

The DHFR stock solution included: 100 μM DHFR, 500 μM NADPH (DHFR substrate) in Enzo assay buffer X1. The Proteostat was diluted 1:200 in an assay buffer to a Proteostat X5 concentration.

Then, 30 μl 4PBA/TMP, 10 μl DHFR+NADPH and 10 μl Proteostat X5 were mixed to the final reaction mixes: 10 mM/2 mM/400 μM/80 μM/0 μM 4PBA/100 μM TMP + 20 μM DHFR + 100 μM NADPH + Proteostat X1.

40 μl of each of these reactions were transferred into a 96‐well plate, and thermal shift assay was conducted using a melting curve program of Bio‐Rad CFX196 qPCR machine (25–95°C, 0.5°C increments, 30 s/°C, HEX channel).

The experiment included background controls for all samples (the same mixtures and concentrations, without the DHFR). Once fluorescence reads were obtained, the background reads were subtracted from the experimental reads. Then, the reads were normalized to the maximum read in each condition. In addition, the experiment was done using 2 DHFRs, from the organisms: *Xanthomonas axonopodis* and *Clostridium beijerinckii* and gave very similar results. Therefore, to avoid redundancy, the manuscript includes only the *Xanthomonas axonopodis* DHFR data.

### 6.10 SYPRO Orange thermal shift aggregation assay

To measure thermal stability in 4PBA presence, it was tested in a thermal shift assay using SYPRO Orange (catalog number: S5692, Sigma‐Aldrich). First, the k‐p assay buffer was freshly prepared (25 mM potassium phosphate pH 7.8, 1 mM EDTA, 1 mM DTT). Then, 16.67 mM 4PBA initial stock was prepared by diluting 4PBA powder in k‐p assay buffer and titrating with an equimolar amount of NaOH. The initial stock was diluted to the following concentrations: 16.67 mM, 3.33, 0.67, 0.13, 0 mM. trimethoprim (TMP) is an inhibitor of DHFR and was used as a positive control (diluted in k‐p assay buffer).

The DHFR stock solution included: 200 μM DHFR, 1000 μM NADPH (DHFR substrate) in k‐p assay buffer. The SYPRO Orange was diluted 1:100 in an assay buffer to a SYPRO Orange X50 concentration.

Then, 30 μl 4PBA/TMP, 10 μl DHFR+NADPH, and 10 μl SYPRO Orange X50 were mixed to the final reaction mixes: 10 mM/2 mM/400 μM/80 μM/0 μM 4PBA/100 μM TMP + 40 μM DHFR + 200 μM NADPH + SYPRO Orange X10.

30 μl of each of these reactions were transferred into a 96‐well plate, and thermal shift assay was conducted using a melting curve program of Bio‐Rad CFX196 qPCR machine (25–95°C, 0.5°C increments, 30 s/°C, HEX channel).

The experiment included background controls for all samples (the same mixtures and concentrations, without the DHFR). Once fluorescence reads were obtained, the background reads were subtracted from the experimental reads. Then, the reads were normalized to the maximum read in each condition. In addition, the experiment was done using 2 DHFRs, from the organisms: *Xanthomonas axonopodis* and *Clostridium beijerinckii* and gave very similar results. Therefore, to avoid redundancy, the manuscript includes only the *Xanthomonas axonopodis* DHFR data.

### 6.11 4PBA and thapsigargin ER stress assays

Media of different 4PBA concentrations were prepared as mentioned above (see *“4PBA SUnSET assays in cells”* section). Then, medium was changed to the corresponding 4PBA treatment for 2 h, and thapsigargin (Alomone labs, catalog number: T‐650) was added directly into the medium to a final concentration of 100 nM for additional 6 h (in total 8 h of 4PBA, out of which 6 h are with 100 nM thapsigargin). Where DMSO is indicated, a similar volume of DMSO (Sigma‐Aldrich, catalog number: D9170) was added instead of thapsigargin.

### 6.12 Antibody list

**Table:** 

Antibody	Company	Catalog number	Dilution
*Renilla* Luciferase	Invitrogen	PA5‐32210	1:1000
Myc‐Tag (9B11)	Cell Signaling Technology	2276 S	1:1000
5‐fluorouridine (BrdU clone BU‐33)	Sigma‐Aldrich	B8434‐100UL	1:300
4 E‐BP1 total	Cell Signaling Technology	9644	1:1000
4 E‐BP1 phosphorylation (Ser65)	Cell Signaling Technology	9451	1:1000
GFP	Roche	47,859,600	1:2000
Puromycin	Sigma‐Aldrich	MABE343	1:20,000
BiP (C50B12)	Cell Signaling Technology	3177	1:1000
eIF2α phosphorylation	Cell Signaling Technology	9721	1:1000
GADD34	Thermo Fisher Scientific	PA1139	1:1000
eIF2α total	Cell Signaling Technology	9722	1:1000
XBP1	Novus Biologicals	NBP1‐77681SS	1:1000
HSC70 (B‐6)	Santa Cruz Biotechnology	SC‐7298	1:1000
β‐Actin	Cell Signaling Technology	4970	1:1000
HSPA2	Sigma‐Aldrich	HPA000798	1:1000
Goat anti‐mouse IgG H + L Alexa Fluor Plus 488	Invitrogen	A32723	1:200
Rabbit anti‐mouse IgG H&L (HRP)	abcam	Ab97046	1:10,000
Goat anti‐rabbit IgG H&L (HRP)	abcam	Ab6721	1:10,000

### 6.13 RNA purification and cDNA reverse transcription

RNA was purified using EZ‐RNA II Kit (Biological Industries Israel, catalog number: 20‐410‐100), according to the manufacturer's protocol.

Then, cDNA was reverse‐transcribed using qScript® cDNA Synthesis Kit (QIAGEN, catalog number: 95047), according to manufacturer's protocol, with 1000 ng RNA per sample.

### 6.14 Semi‐quantitative PCR

PCR was done using Phire Green Hot Start II PCR Master Mix enzyme (Thermo Fisher Scientific, catalog number: F126S), according to manufacturer's recommendations. The primers and number of cycles (verified to be below the maximal amplification) for each gene were as follows:

**Table:** 

Transcript	Fw primer	Rev primer	Cy number
XBP1u/s	CCTTGTAGTTGAGAACCAGG	GGGGCTTGGTATATATGTGG	30
β‐Actin	AGCACAGAGCCTCGCCTTT	CGCGGCGATATCATCATCCA	26

PCR products were electrophoresed on a 3.2% gel.

## AUTHOR CONTRIBUTIONS

DT and DS conceived, led, planned, and designed the study. DS, ZS, BT, and ME performed the biochemical experiments. DS performed the cell culture and molecular biology experiments. BA and SB provided help with the biochemical experiments and designing and planning them. DT and DS wrote the manuscript.

## FUNDING INFORMATION

This work was supported by the David and Inez Myers Foundation, and by the Bio‐tech and Negev fellowships of Kreitman School of Advanced Research of Ben Gurion University.

## CONFLICT OF INTEREST

The authors declare no conflicting or competing interests.

## Data Availability

All the data are present in the manuscript.
